# Supplementary material for: Examination of Microcystin Adsorption by the Type of Plastic Materials Used during the Procedure of Microcystin Analysis
Source: Toxins (Basel). 2022 Sep 7;14(9):625. doi: 10.3390/toxins14090625 (PMC9502409; doi:10.3390/toxins14090625)
Supplement: Supplementary file 1 [file toxins-14-00625-s001.zip › toxins-1870009-supplementary.pdf]

toxins

MDPI

Article

Examination of Microcystin Adsorption by the Type of Plastic Materials Used during the Procedure of Microcystin Analysis

Chan Seo [1], Joo Won Lee [2], Won-Kyo Jung [3,4], Yoon-Mi Lee [5], Seungjun Lee [1] and Sang Gil Lee [1,2,*]

[1] Department of Food Science and Nutrition, Pukyong National University, Nam-Gu, Busan 48513, Korea
[2] Department of Smart Green Technology Engineering, Pukyong National University, Busan 48513, Korea
[3] Research Center for Marine Integrated Bionics Technology, Pukyong National University, Busan 48513, Korea
[4] Department of Biomedical Engineering, Pukyong National University, Busan 48513, Korea
[5] Food Safety and Processing Research Division, National Institute Fisheries Science, Busan 46083, Korea
* Correspondence: sglee1125@pknu.ac.kr

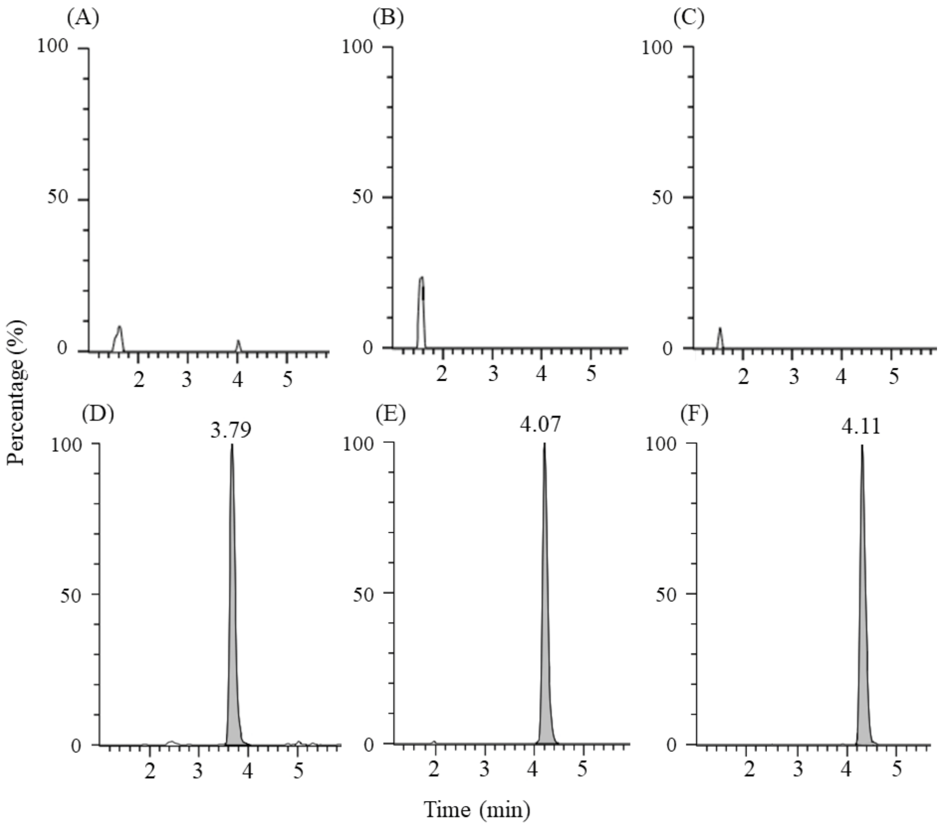

Figure S1. Multiple reaction monitoring chromatograms of (**A**) blank for MC-RR, (**B**) blank for MC-LR, (**C**) blank for MC-YR, (**D**) MC-RR, (**E**) MC-LR, and (**F**) MC-YR.

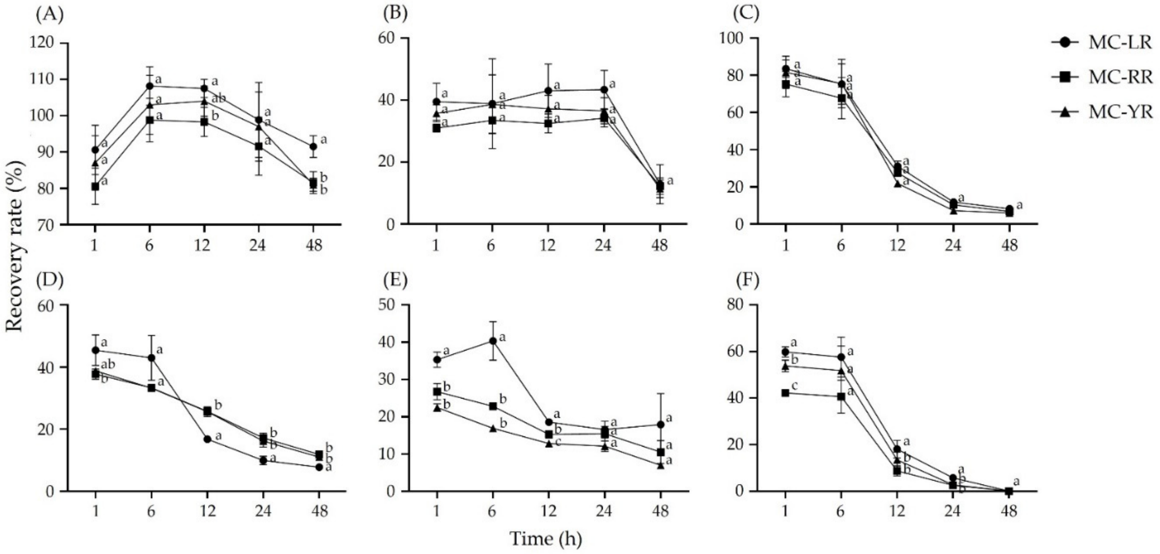

Figure S2. Recovery rates of three MC variants by a given six plastic storage container. (**A**) PET; (**B**) PP; (**C**) PFA; (**D**) LDPE; (**E**) HDPE; and (**F**) PS. Different letters (a-e) indicate significant difference ($p < 0.05$).
